# Hydrogen gas with hypothermic machine perfusion induces lipidomic alterations in donation-after-cardiac-death rat livers

**DOI:** 10.1016/j.jlr.2026.101040

**Published:** 2026-04-16

**Authors:** Yusuke Minami, Siddabasave Gowda B. B. Gowda, Kengo Shibata, Sodai Sakamoto, Divyavani Gowda, Tsuyoshi Shimamura, Akinobu Taketomi, Hitoshi Chiba, Moto Fukai, Shu-Ping Hui

**Affiliations:** 1 Graduate School of Health Sciences, Hokkaido University, Kita-ku, Sapporo, Japan; 2 Faculty of Health Sciences, Hokkaido University, Kita-ku, Sapporo, Japan; 3 Graduate School of Global Food Resources, Hokkaido University, Kita-Ku, Sapporo, Japan; 4 Graduate School of Medicine, Gastroenterological Surgery 1, Hokkaido University, Kita-ku, Sapporo, Japan; 5 Division of Organ Transplantation, Hokkaido University Hospital, Kita-ku, Sapporo, Japan; 6 Department of Nutrition, Sapporo University of Health Sciences, Higashi-ku, Sapporo, Japan; 7 Graduate School of Well-being, Department of Food Environment, Fuji Women’s University, Ishikari, Japan

**Keywords:** ischemia reperfusion injury, lipid metabolism, lipidomics, lysophospholipid, mitochondria, phosphatidic acid, phospholipids

## Abstract

Hypothermic machine perfusion (HMP) combined with hydrogen gas has previously been shown to mitigate ischemia-reperfusion injury (IRI) in rat liver, but without full recovery of function. This study examined how hydrogen gas modulates hepatic lipid metabolism during HMP using donation after cardiac death (DCD) rat livers. Untargeted liquid chromatography-mass spectrometry was employed to perform comprehensive lipidomic profiling of liver samples. The analysis results revealed distinct lipid metabolic alterations across cold storage, machine perfusion (MP), and hydrogen-supplemented perfusion (MP-H2) groups compared to healthy controls. Compared with MP, MP-H2 treatment reduced lysophosphatidylinositol (LPI) levels and the LPI/phosphatidylinositol ratio while increasing phosphatidic acid (PA) species such as PA (18:0/18:1) and PA (18:0/20:4). Elevated lysophosphatidylethanolamine and ceramide in MP-H2 suggested adaptive remodeling of membrane lipids. The ratio of monolysocardiolipin to cardiolipin increased in the MP group, but was reduced following hydrogen gas treatment. These lipidomic shifts imply that hydrogen gas attenuates IRI by stabilizing lipid homeostasis and may improve DCD graft viability. Furthermore, the restoration of key lipid species associated with mitochondrial integrity and membrane remodeling suggests that hydrogen gas supports bioenergetic recovery and limits oxidative membrane damage during reperfusion. Overall, these findings highlight the potential of hydrogen-enriched perfusion as a metabolic intervention to enhance organ preservation, reduce mitochondrial dysfunction, and extend the useable lifespan of DCD liver grafts for transplantation.

Liver transplantation remains the only curative treatment for patients with end-stage liver disease; however, the global shortage of donor organs continues to limit its availability (1). To alleviate this shortage, donation after cardiac death (DCD) has gained increasing attention as an alternative source of donor livers (2). Despite this potential, DCD livers are highly susceptible to ischemia-reperfusion injury (IRI), and no standardized method currently ensures their safe clinical use (3, 4). IRI refers to the tissue damage that occurs when blood supply returns to a previously ischemic organ, with the reperfusion phase exacerbating the injury caused by ischemia (5). In DCD livers, IRI develops through three stages: warm ischemia due to cardiac arrest, cold ischemia during storage, and reperfusion accompanied by reoxygenation (3). The pathogenesis of IRI primarily involves the excessive formation of reactive oxygen species (ROS), leading to oxidative stress (6). Elevated ROS levels trigger lipid peroxidation, mitochondrial dysfunction, inflammation, and apoptosis, ultimately resulting in hepatocellular injury (7, 8). Minimizing IRI is therefore a critical goal for improving transplantation outcomes. Cold storage (CS) has long been considered the gold standard for organ preservation, but it carries significant risks of IRI, particularly in DCD donor livers (1). Mechanical perfusion (MP) has emerged as a promising alternative, as it circulates oxygenated perfusate to sustain cellular metabolism and mitigate ischemic damage (9, 10). Hypothermic machine perfusion (HMP) further suppresses mitochondrial activity, thereby protecting subcellular structures such as mitochondria and nuclei (11). Nevertheless, there is no consensus regarding the optimal perfusion protocol, and standardized guidelines have yet to be established (12).

Recent studies have demonstrated that combining HMP with hydrogen gas treatment preserves mitochondrial cristae structure and reduces the apoptotic index in a rat model of hepatic IRI, compared with HMP alone. However, key functional metrics such as portal vein resistance and bile secretion were not fully restored, suggesting the need for further refinement before clinical translation (13). The mechanism underlying the partial improvement observed with hydrogen supplementation remains unclear. Hydrogen gas administered during reperfusion suppresses cytoplasmic MKK4–JNK-mediated cell death signaling and preserves mitochondrial function in DCD rat livers. This is proposed to interrupt the vicious cycle of mitochondrial dysfunction and oxidative stress, thereby mitigating IRI (3). Given that hepatic IRI is closely associated with lipid metabolic disruption (14), understanding these lipid alterations may provide insights into hydrogen’s protective role. Oxidative stress contributes to lipid peroxidation (15), while ischemia and reperfusion stimulate phospholipid (PL) hydrolysis, generating lysophospholipids (Lyso-PLs) and FFAs such as arachidonic acid, which possess proinflammatory properties (16). Attenuation of IRI by hydrogen gas administration during HMP may be closely associated with modulation of the lipid metabolic profile. To elucidate the metabolic basis of hydrogen-mediated protection, we conducted a comprehensive lipidomic analysis using high-performance liquid chromatography coupled to linear ion trap–Orbitrap mass spectrometry (HPLC/LTQ-Orbitrap-MS). This study compares hepatic lipid profiles among CS, MP, and hydrogen-supplemented MP (MP-H2) conditions to clarify how hydrogen gas modulates lipid metabolism and mitigates IRI during HMP.

## Materials and Methods

### Materials

Blezer UW (CS solution) and Blezer MPS (mechanical perfusion solution) were purchased from Bridge to Life (Northbrook, IL). Pure H_2_, pure CO_2_, and mixed gas (95% O_2_ and 5% CO_2_) were obtained from AIR WATER, Inc. (Osaka, Japan). LC/MS grade solvents such as methanol, chloroform, and isopropanol were purchased from Wako Pure Chemical Industries, Ltd (Osaka, Japan). A 1 mol/l ammonium acetate solution used as the HPLC solvent was purchased from Kanto Chemical Co. (Tokyo, Japan). Ceramic beads (1.4 mm) for sample homogenization were obtained from Thermo Fisher Scientific (Tokyo, Japan). EquiSPLASH LIPIDOMIX and oleic acid-d9, used as internal standards, were purchased from Avanti Polar Lipids, Inc. (Alabaster, AL). IS solution was prepared consisting of oleic acid-d9 (10 μg/ml) and EquiSPLASH LIPIDOMIX mixtures (1 μg/ml) in methanol. Cytosolic phospholipase A2 (cPLA2) Assay Kit was purchased from Cayman Chemical (Item No. 765021). 20 × TBS (pH 7.4) was purchased from Nippon Gene (Toyama, Japan).

### Animals and sample collection

Animal experiments were conducted in accordance with the Guidelines for the Care and Use of Laboratory Animals and were approved by the Animal Experimentation Committee of Hokkaido University. Male Wistar rats (8–10 weeks old) were obtained from Sankyo Labo Service Corporation, Inc. (Tokyo, Japan). The animals were housed under standard conditions with free access to laboratory chow (Oriental Yeast Co., Tokyo, Japan) and tap water.

Liver resection from DCD rats was performed as previously described (3). Briefly, rats were anesthetized with inhaled isoflurane without prior fasting. The abdominal aorta and common bile duct were cannulated using an 18 G Teflon catheter (Nipro, Osaka, Japan) and BD Intramedic polyethylene tubing PE-10 (Thermo Fisher Scientific, NJ), respectively. Cardiopulmonary arrest was induced by diaphragmatic incision, followed by exsanguination achieved through clamping and proximal transection of the descending aorta. The abdominal cavity was rinsed twice with warm saline (37°C). Thirty minutes after cardiac arrest, the liver was flushed with 60 ml of ice-cold saline via the abdominal aorta, followed by perfusion with 25 ml of ice-cold Belzer-UW or Belzer-MPS solution through the portal vein.

Rat liver grafts were allocated into four experimental groups. In the control group (n = 6), grafts were excised without inducing cardiac arrest and immediately reperfused using the isolated perfused rat liver (IPRL) system described below. In the CS group (n = 6), DCD grafts were preserved in Belzer-UW solution at 4°C for 3 h prior to reperfusion. In the machine perfusion (MP) group (n = 9), DCD grafts underwent HMP in Belzer-MPS at 7°C for 3 h, followed by reperfusion. In the MP-H2, n = 6, HMP was performed as in the MP group, but with Belzer-MPS continuously saturated with hydrogen gas at a concentration of 4% of the total flow rate during perfusion.

**Fig 1:**
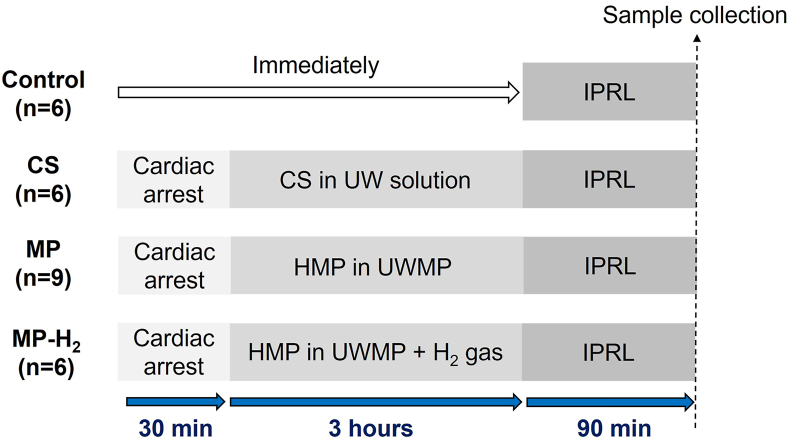
Protocol for creating experimental groups. CS, cold storage; HMP, hypothermic machine perfusion; IPRL, isolated perfused rat liver.

The HMP and IPRL systems incorporated identical equipment setups. For reperfusion, 300 ml of Krebs–Henseleit bicarbonate buffer containing sodium cholate was used to perfuse the livers for 90 min at 37°C, with flow rates maintained at 2–3 ml/min/g liver. Perfusion pressure was set at 8 cm H2O for the control group and 12 cm H2O for the MP group, while oxygen partial pressure was maintained within the optimal range of 450–550 mmHg. The protocol for creating the experimental group is shown in Figure 1. Following IPRL, the livers were weighed and immediately stored at 80°C until lipid extraction.

### Lipid extraction

Approximately, 100 mg of rat liver tissue was weighed and transferred into a 2 ml microtube. The samples were homogenized twice for 30 s using ceramic beads and a bead homogenizer. Then, 1 ml of methanol was added, and the mixture was further homogenized (30 s × 2 cycles). Total lipids were extracted using a modified single-phase Bligh and Dyer method (17). Briefly, 100 μl of the homogenate (equivalent to 10 mg tissue) was transferred into a 1.5 ml microtube. An internal standard solution (100 μl, prepared in methanol; refer to the 2.1. Materials section for details) was added and vortexed at 3,500 rpm for 1 min. Subsequently, 100 μl of chloroform and 20 μl of Milli-Q water were added, followed by vortexing at 3,500 rpm for 5 min. The mixture was centrifuged at 15,000 rpm for 10 min, and the supernatant was collected in a new 1.5 ml microtube. Re-extraction was performed by adding 200 μl of methanol, 100 μl of chloroform, and 20 μl of Milli-Q water to the remaining pellet, followed by vortexing and centrifugation under the same conditions. The two supernatants were combined and evaporated to dryness using a centrifugal evaporator under vacuum at 4°C. The dried lipid extract was reconstituted in 100 μl of methanol, vortexed at 3,500 rpm for 1 min, and centrifuged at 15,000 rpm for 10 min at 4°C. The final supernatant was transferred to an LC/MS vial for analysis.

### LC/MS analysis

For lipid separation, an HPLC system (Shimadzu Corporation, Kyoto, Japan) equipped with an Atlantis T3 column (3 μm, 2.1 × 150 mm; Waters, Milford) was used. The column oven was maintained at 40°C, and the mobile phase flow rate was set at 200 μl/min. The mobile phase consisted of 10 mM ammonium acetate (solvent A), isopropanol (solvent B), and methanol (solvent C). Gradient elution was performed in both positive and negative ion modes following previously reported conditions (18). The injection volume was 10 μl. Mass spectrometric analysis was carried out using an LTQ Orbitrap mass spectrometer (Thermo Fisher Scientific Inc., San Jose, CA) operating in both positive and negative ionization modes under the same parameters as previously described (19). We performed quality control runs during each batch submission and continuously monitored the LC/MS operating conditions. The acquired raw data were processed using MS-DIAL software (version 5.1; https://systemsomicslab.github.io/compms/msdial/main.html) with parameter settings identical to those in a previous study (20) for lipid annotation and peak area quantification. Lipid molecular species were identified by MS and MS/MS spectra using Xcalibur version 2.2 (https://www.thermofisher.com) (Thermo Fisher Scientific, Waltham). For semiquantitative analysis, internal standards corresponding to the same lipid class or a closely related subclass were used. The analyte concentrations were determined by multiplying the peak area ratio of the analyte to the internal standard by the amount of internal standard added. All data were normalized to the tissue weight used for extraction.

### Measurement of total phosphatidic acid A2 activity

Livers were weighed and transferred to 2 ml tubes, followed by the addition of 1 ml of 1× TBS (pH 7.4) per 100 mg of tissue. Five ceramic beads were added, and the samples were homogenized for 30 s. After centrifugation at 4°C for 15 min at 10,000 rpm, the supernatants were collected into new Eppendorf tubes and stored at 80°C until assay. Total phosphatidic acid A2 (PLA2) activity assay was performed using a commercial cPLA2 assay kit (Cayman Chemical, Item No. 765021) according to the manufacturer’s instructions. Absorbance at 414 nm was measured using a BIO-RAD xMark™ microplate spectrophotometer.

### Statistical analysis

Sparse partial least squares–discriminant analysis and hierarchical clustering correlation analysis were performed using MetaboAnalyst version 6.0 (https://www.metaboanalyst.ca/MetaboAnalyst/ModuleView.xhtml). Data visualization and statistical analyses were conducted with GraphPad Prism version 8 (https://www.graphpad.com/). All results are presented as mean ± SD. ANOVA followed by Tukey’s multiple comparison test was applied to assess statistical significance between groups, with *P* < 0.05 considered significant.

## Results

### Lipid fingerprint and multivariate analysis of lipid metabolites

**Fig 2:**
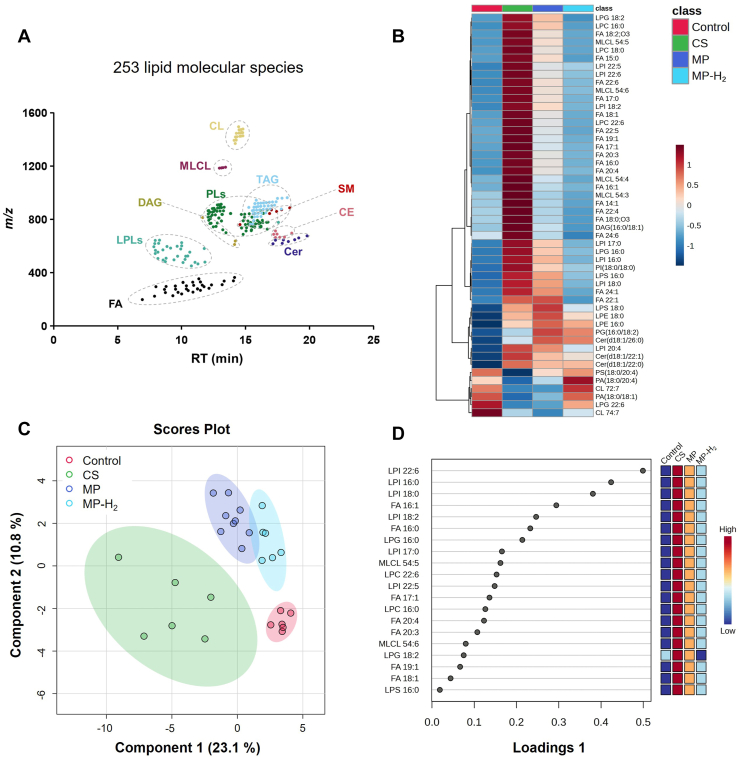
Lipid profile analysis. A: Lipid molecular species detected and m/z versus RT elution profiles. B: Hierarchical cluster correlation analysis of the top 50 significantly changed lipids (*P* < 0.05, clustering method: ward, distance measure: Euclidean). C: Sparse partial least squares discriminant analysis (sPLS-DA score plot). D: Sparse partial least squares discriminant analysis (sPLS-DA) loading plot of component1. Control (n = 6), CS (n = 6), MP (n = 9), and MP-H_2_ (n = 6). CE, cholesteryl ester; Cer, ceramide; CL, cardiolipin; DAG, diacylglycerol; FA, fatty acid; LPLs, lysophospholipids; MLCL, monolysocaldiolipin; PLs, phospholipids; SM, sphingomyelin; TAG, triacylglycerol.

Lipid profiles in cold ischemia–reperfusion rat liver models were analyzed using HPLC/LTQ-Orbitrap-MS in both positive and negative ionization modes. The overall lipid elution profile (*m/z* vs. retention time) is presented in Figure 2A. Lipidomic analysis identified a total of 253 lipid molecular species spanning multiple lipid classes. A comprehensive list of these metabolites and their quantified concentrations is provided in Supplemental Table S1. Hierarchical cluster correlation analysis of the top 50 lipid species exhibiting significant intergroup differences is shown in Figure 2B. In the heatmap, red regions represent higher relative abundance, while blue regions indicate lower abundance. Strong red coloration was notably observed in the CS group, followed by MP, whereas the MP-H2 group displayed a pattern more closely resembling the control. This gradient suggests that the deviation of lipid composition from the control decreased in the order CS > MP > MP-H2. Among the top 50 altered lipid species, FFAs and Lyso-PLs,including LPE, lysophosphatidylserine (LPS), lysophosphatidylglycerol (LPG), lysophosphatidylinositol (LPI), lysophosphatidylcholine (LPC), and monolysocardiolipin (MLCL),were the major contributors. In contrast, the MP-H2 group exhibited relatively higher levels of several ceramide (Cer) species, such as Cer(d18:1/26:0), Cer(d18:1/22:1), and Cer(d18:1/22:0).

Sparse partial least squares–discriminant analysis was conducted on all 253 detected lipid species (Fig. 2C). Component 1 and component 2 accounted for 23.1% and 10.8% of the total model variance, respectively. The four experimental groups were clearly separated, with Component 1 primarily distinguishing CS and MP from Control and MP-H2. The loading plot (Fig. 2D) revealed that this separation was predominantly driven by lyso-PLs and FFAs, particularly LPI species such as LPI 22:6, LPI 16:0, LPI 18:0, LPI 18:2, LPI 17:0, and LPI 22:5.

### Hierarchical cluster analysis of lipids with distinct group differences

**Fig 3:**
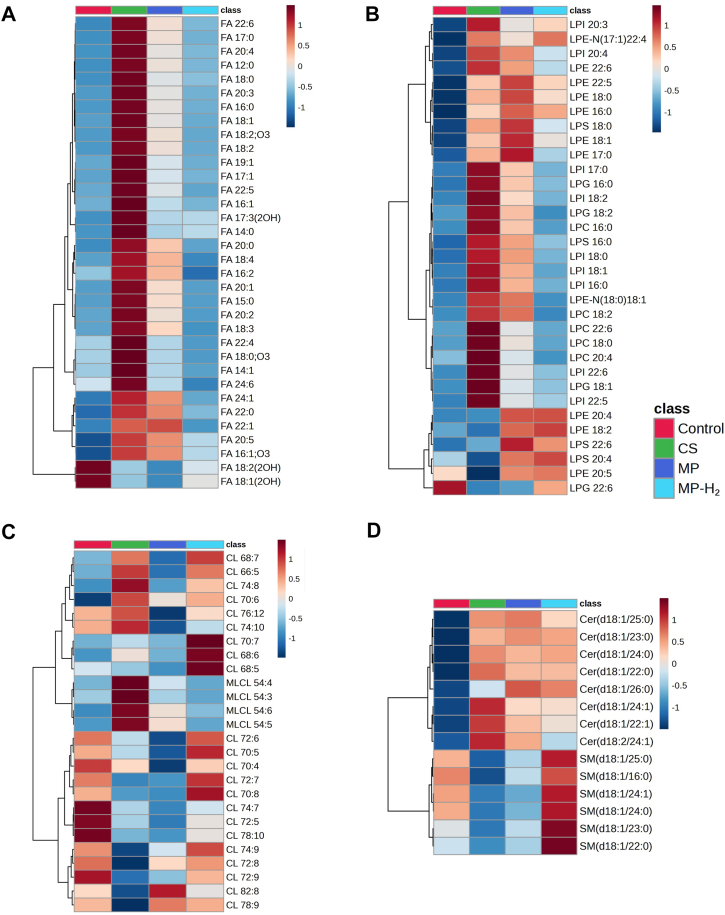
Hierarchical cluster analysis of lipids with characteristic trends among groups. A: FFA. B: LPC, LPE, LPG, LPS, and LPI. C: CL and MLCL. D: sphingolipid, (clustering method: ward, distance measure: Euclidean). Control (n = 6), CS (n = 6), MP (n = 9), and MP-H_2_ (n = 6). CL, cardiolipin; CS, cold storage; LPC, lysophosphatidylcholine; LPE, lysophosphatidylethanolamine; LPG, lysophosphatidylglycerol; LPI, lysophosphatidylinositol; LPL, lysophospholipid; LPS, lysophosphatidylserine; MLCL, monolysocardiolipin; MP, machine perfusion; MP-H_2_, hydrogen-supplemented MP.

Hierarchical clustering heatmaps were generated to visualize relative lipid abundance patterns across groups. The distributions of FFAs and Lyso-PLs, including LPC, LPE, LPG, LPI, and LPS, are presented in Figure 3A, B, respectively. Among these lipid species, FFAs showed the highest accumulation in the CS group, followed by MP, while levels in MP-H2 nearly returned to those observed in the control group. This trend suggests that hydrogen supplementation during HMP effectively mitigated FFA accumulation, which is typically indicative of membrane degradation and lipolytic activity during ischemia–reperfusion stress. A similar pattern was observed for Lyso-PLs, which are key intermediates of PL hydrolysis. The CS group exhibited the greatest relative abundance of Lyso-PLs, whereas these levels progressively decreased in MP and further in MP-H2. In the MP-H2 group, several LPE species (LPE 18:1, LPE 17:0, LPE 18:0, LPE 16:0, LPE 22:5, LPE 18:2, LPE 20:5, and LPE 20:4) remained moderately elevated compared with control levels, along with selected LPS (LPS 18:0, LPS 20:4, LPS 22:6) and LPG 22:6 species. These findings imply that hydrogen gas may not completely suppress PL remodeling but instead modulates the balance between Lyso-PL formation and reacylation, reflecting adaptive membrane repair processes.

Heatmaps for cardiolipin (CL), MLCL, Cer, and sphingomyelin (SM) profiles are displayed in Figure 3C, D. MLCL species were markedly abundant in the CS group, consistent with mitochondrial membrane degradation under severe ischemic stress. CLs with long total acyl chain carbons (>70) were most enriched in the control group, suggesting preservation of mature mitochondrial lipid composition under physiological conditions. In contrast, these CL species were reduced in all ischemic or perfusion-treated groups, indicating partial loss of mitochondrial membrane integrity. Cer levels were low in the control group but substantially increased in CS, MP, and MP-H2, suggesting activation of sphingolipid-mediated stress responses. Notably, the moderate Cer accumulation observed in MP-H2 compared with CS suggests that hydrogen-enriched perfusion partially alleviates sphingolipid-associated apoptotic signaling. Conversely, SM exhibited increased levels in the MP-H2 group compared with both CS and MP groups. Collectively, these hierarchical clustering results highlight distinct lipid remodeling patterns across groups and demonstrate that hydrogen supplementation during HMP promotes partial recovery of lipid homeostasis in DCD rat livers.

### Volcano plot analysis

**Fig 4:**
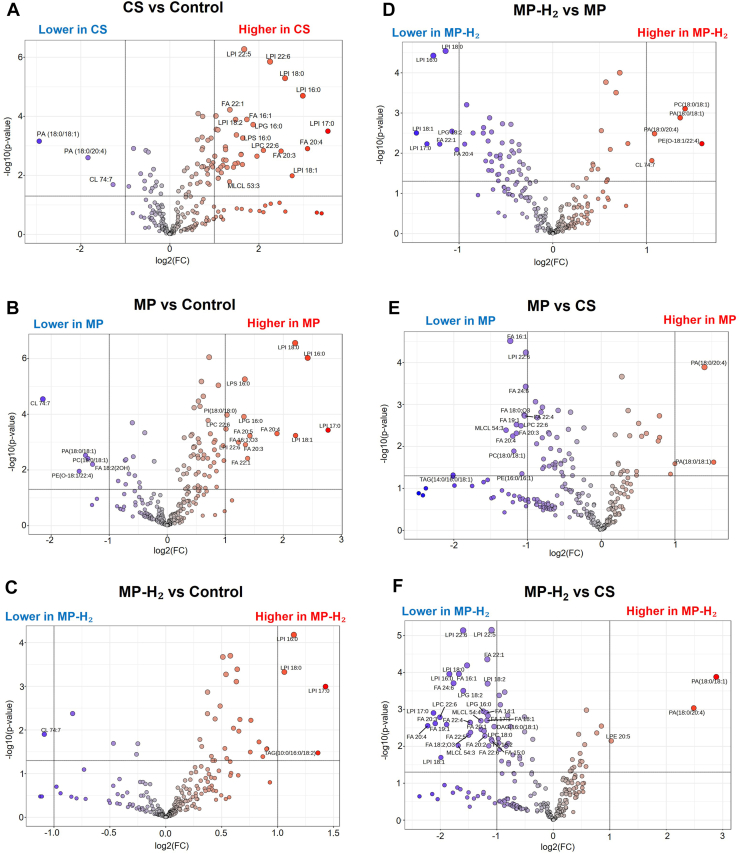
Volcano plots showing significantly altered lipids in cold ischemia and reperfusion (*t* test, *P*< 0.05). A: CS versus Control group. B: MP versus Control group. C: MP-H_2_ versus Control group. D: MP-H_2_ versus MP. E: MP versus CS. F: MP-H_2_ versus CS. CS, cold storage; MP, machine perfusion; MP-H_2_, hydrogen-supplemented MP.

Volcano plot analyses comparing the control and each experimental group are shown in Figure 4A–C. In these plots, the x-axis represents the fold change (log2 scale), while the y-axis indicates the statistical significance (−log10 *P*-value). Lipid molecular species with the most pronounced changes, those exhibiting high variability and strong statistical significance, appear in the upper right or upper left corners of the plots.

In the CS group, extensive alterations in the hepatic lipidome were observed compared with the control. Lyso-PLs (particularly, LPI) and FFAs were significantly elevated, highlighting significant membrane PL turnover and enhanced lipolytic activity during cold ischemia. Eight LPI molecular species exhibited more than a twofold increase, including LPI 16:0, LPI 17:0, LPI 18:0, LPI 18:1, LPI 18:2, LPI 20:3, LPI 22:5, and LPI 22:6, suggesting possible activation of PLA2-mediated hydrolysis of phosphatidylinositol (PI). In contrast, several phosphatidic acid (PA) species, such as PA (18:0/18:1) and PA (18:0/20:4), along with CL (CL 74:7), were significantly reduced, indicating suppression of PL biosynthesis and mitochondrial membrane degradation under ischemic stress. The MP group also demonstrated increased levels of six LPI species (LPI 16:0, LPI 17:0, LPI 18:0, LPI 18:1, LPI 20:4, and LPI 22:6) and FFAs relative to the control, though the fold changes were smaller than those in the CS group. This attenuation in lipid perturbation implies that HMP partially preserved lipid homeostasis and mitigated ischemia-induced degradation.

In contrast, the MP-H2 group showed a significantly reduced extent of lipid alteration compared with both CS and MP groups. Only three LPI species (LPI 16:0, LPI 17:0, and LPI 18:0) were elevated, while saturated fatty acid–enriched species predominated. A concurrent reduction in CL 74:7 was observed. A direct comparison between MP and MP-H2, shown in Figure 4D, further clarifies the metabolic effects of hydrogen during perfusion. Hydrogen supplementation increased several lipid species, including PA (18:0/18:1), PA (18:0/24:0), CL 74:7, phosphatidylcholine (PC 18:0/18:1), and phosphatidylethanolamine (PE(O-18:1/22:4)), indicating enhanced PL remodeling and mitochondrial membrane recovery. Conversely, levels of FFAs; four key LPI species (LPI 16:0, LPI 17:0, LPI 18:0, LPI 18:1); and LPG 18:2 significantly decreased compared with MP alone. Volcano plots comparing CS with MP, and CS with MP-H2, are presented in Figure 4E, F, respectively. Similar patterns were observed in both figures, with PA (18:0/18:1) and PA (18:0/20:4) showing significantly higher abundance compared to CS. These changes suggest that hydrogen gas not only suppresses PL hydrolysis but may also promote the reacylation process, thereby restoring structural lipids involved in membrane integrity and energy metabolism. Overall, the volcano plot analyses demonstrate a stepwise improvement in lipid stabilization from CS to MP to MP-H2, supporting the protective metabolic role of hydrogen gas in maintaining hepatic lipid homeostasis under ischemia–reperfusion conditions.

### Variation in total lipid content and total lyso-PL/total PL at the subclass level

**Fig 5:**
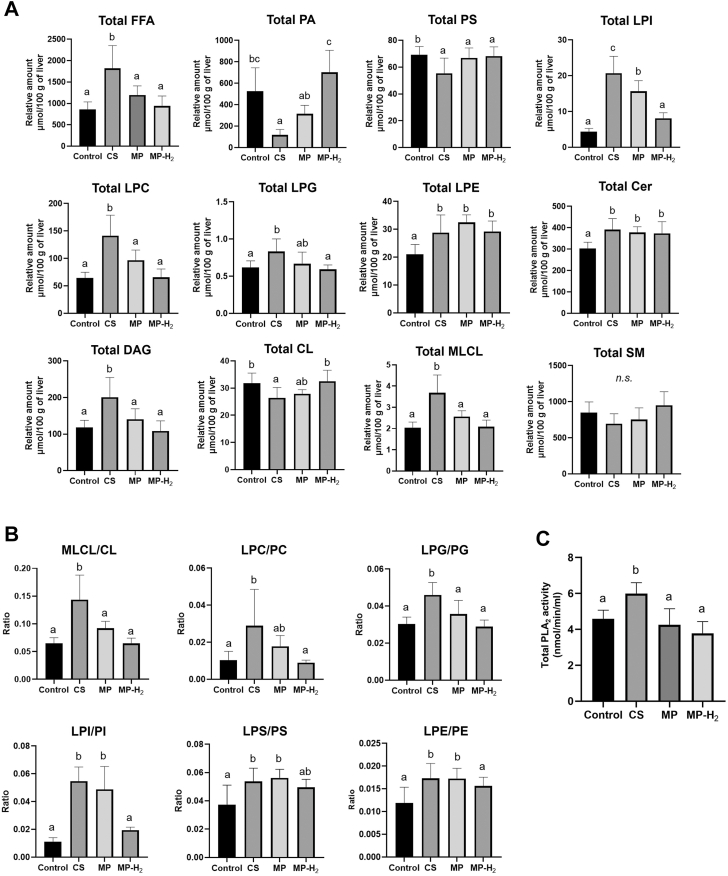
A: The total content of lipid subclass levels. B: Total Lyso-PL/Total PL ratio at subclass level. C: Total PLA2 activity. Data are presented as mean ± standard deviation (SD). One-way ANOVA with Tukey post hoc test. The different letters indicate statistically significant differences, *P* < 0.05, *n.s.* not significant. Control (n = 6), CS (n = 6), MP (n = 9), and MP-H_2_ (n = 6). CS, cold storage; MP, machine perfusion; MP-H_2_, hydrogen-supplemented MP; PL, phospholipid; PLA2, phospholipase A2.

The total lipid content across major subclasses exhibiting significant intergroup differences is presented in Figure 5A. Distinct shifts in lipid subclass composition were observed among the experimental groups, reflecting the differential impact of CS, MP, and MP-H2 on hepatic lipid metabolism. In the CS group, several lipid subclasses showed pronounced alterations relative to the control, including increases in FFAs, LPC, LPG, DAG, CL, and MLCL. Elevated FFAs and Lyso-PLs suggest enhanced phospholipase activity and membrane breakdown as a consequence of ischemic stress. The accumulation of DAG further indicates activation of lipid remodeling and potential involvement in signaling pathways associated with cell damage. Meanwhile, the reduction in total CL accompanied by increased MLCL content implies mitochondrial membrane remodeling or degradation, consistent with oxidative injury to CL during ischemia-reperfusion. Interestingly, MP-H2 induced distinct changes in PA and LPI levels compared with both CS and MP. The increase in PA species such as PA (18:0/18:1) and PA (18:0/20:4) suggests enhanced lipid biosynthetic recovery and membrane repair through reactivation of the glycerophospholipid pathway. Concurrently, the marked decrease in LPI implies suppression of PL hydrolysis and attenuation of inflammatory lipid signaling. These trends indicate that hydrogen gas may facilitate metabolic re-equilibration of lipid turnover processes during HMP. Among all subclasses, LPE and Cer levels were significantly elevated in every treatment group compared to the control, with no significant differences among CS, MP, and MP-H2. Total SM levels did not differ significantly in the MP-H2 group compared with the other groups; however, they tended to approach levels comparable to those observed in the control group. Collectively, subclass-level lipid analysis reveals that while CS induces extensive lipid degradation, MP mitigates these effects, and hydrogen-enriched perfusion further promotes restoration of lipid homeostasis, particularly through modulation of PA and LPI metabolism.

To evaluate membrane remodeling and lipid turnover, the total Lyso-PL-to-PL ratio was calculated for each lipid subclass, as shown in Figure 5B. This ratio reflects the balance between PL degradation and reacylation, providing insight into membrane integrity. Total MLCL/CL, LPC/PC, and LPG/PG ratios were significantly elevated only in the CS group compared to the control, indicating extensive PL hydrolysis and mitochondrial membrane damage under cold ischemic conditions. In contrast, both MP and MP-H2 groups maintained these ratios at levels comparable to the control, suggesting effective preservation of lipid structural stability during perfusion. The total LPI/PI, LPS/PS, and LPE/PE ratios showed similar patterns. These ratios were markedly increased in the CS and MP groups relative to control, signifying ongoing Lyso-PLs accumulation and stress-induced PL breakdown. However, in the MP-H2 group, these ratios closely approximated those of the control, demonstrating reduced lipid hydrolysis and better maintenance of membrane composition. Notably, significant reductions in total LPI/PI and total LPE/PE ratios were observed in MP-H2 compared to MP alone, reinforcing that hydrogen supplementation effectively suppressed excessive PL turnover and supported restoration of membrane lipid equilibrium. Figure 5C shows the total PLA2 activity, a key enzyme responsible for Lyso-PL production. Total PLA2 activity was significantly elevated in the CS group, while it remained at control levels in both the MP and MP-H2 groups.

## Discussion

With the severity of IRI, the accumulation of Lyso-PLs and FFAs increases markedly. Lyso-PLs are generated when one of the two acyl chains of a PL molecule is cleaved, producing a Lyso-PL and a corresponding FFA. This reaction is catalyzed by phospholipase A (PLA), which hydrolyzes the ester bond of PLs (21, 22). Degradation of PLs compromises mitochondrial membrane integrity, triggers mitochondrial permeability transition, and promotes apoptosis through the release of cytochrome c into the cytoplasm (23). Previous studies have shown that inhibition of cPLA2, a key enzyme within the PLA2 family, or suppression of PL hydrolysis can prevent mitochondrial damage and reduce hepatic IRI (24). In the present study, CS significantly enhanced the production of Lyso-PLs and FFAs, indicating extensive PL breakdown under hypoxic and oxidative stress conditions. MP reduced this accumulation, and MP-H2 further suppressed these changes, suggesting that hydrogen gas may mitigate PLA-mediated lipid hydrolysis. Multivariate analysis supported this observation, revealing that Lyso-PLs, particularly LPI, and FFAs were major contributors to the lipidomic differences distinguishing the MP-H2 and control groups from CS and MP groups. An earlier study reported elevated LPI 18:0 concentration in rat livers subjected to warm ischemia (25). LPI acts as an endogenous ligand for the G-protein–coupled receptor 55 (GPR55), whose expression increases in both patients and animal models of liver injury (26, 27). Activation of the LPI–GPR55 signaling axis stimulates the Rho (Ras homolog family)–ROCK (Rho kinase) pathway, a cascade known to promote inflammation, cytoskeletal disruption, and hepatocellular apoptosis. Inhibition of the Rho–ROCK pathway has been shown to alleviate hepatic IRI (28). Therefore, suppression of LPI accumulation in the MP-H2 group suggests that hydrogen gas may attenuate IRI by modulating the LPI–GPR55–Rho–ROCK signaling pathway. Altogether, these findings indicate that hydrogen-enriched HMP helps preserve mitochondrial membrane integrity and reduces lipid hydrolysis-associated signaling cascades. By stabilizing lipid homeostasis and suppressing inflammatory lipid mediators such as LPI, hydrogen gas contributes to the attenuation of IRI and enhances the metabolic resilience of DCD donor livers during preservation.

MP-H2 treatment effectively prevented the accumulation of FFAs and Lyso-PLs compared with CS and MP groups, although several LPE, LPS, and LPG species remained relatively abundant. Under ischemic conditions, FFA accumulation is detrimental and results from enhanced PL degradation, release of FFAs and Lyso-PLs, and impaired PL synthesis (29, 30). Such lipid alterations promote mitochondrial permeability transition and trigger apoptotic signaling (31). The persistence of certain LPE, LPS, and LPG species in MP-H2 may indicate incomplete suppression of lipid remodeling, potentially contributing to partial organ recovery despite hydrogen treatment. The CS group exhibited markedly lower CL and higher MLCL levels than all other groups. CL, a PL unique to the mitochondrial inner membrane, is essential for maintaining mitochondrial structure and bioenergetic function (32). MLCL accumulation reflects CL degradation and indicates impaired mitochondrial membrane stability (33). The elevated MLCL levels in the CS group suggest significant mitochondrial disruption, whereas the abundance of CL species with longer acyl chains (>70 carbons) in the control group may reflect optimal CL remodeling and membrane fluidity, both critical for sustaining mitochondrial function during IRI (34, 35). In this study, extensive accumulation of Lyso-PLs,including MLCL,was observed in the CS group, whereas these alterations were significantly suppressed in the MP and MP-H2 groups, supporting the utility of Lyso-PLs as predictive markers of tissue injury. Cer species were relatively low in the control group but elevated in CS, MP, and MP-H2 groups. As sphingolipids, Cer regulate cell growth, death, and migration, and are known to increase mitochondrial membrane permeability and promote apoptosis (36). Previous studies have reported significant Cer accumulation in the liver following cold IRI (37). Therefore, enhanced Cer levels across the ischemic and perfused groups may contribute to the exacerbation of IRI, highlighting a potential target for further intervention.

Mitochondrial Ca^2+^-independent phospholipase A_2_γ deacylates PC, PE, PG, and CL to generate LPC, LPE, LPG, and MLCL (38). Accumulation of MLCL disrupts CL homeostasis and promotes alterations in mitochondrial cristae structure (39). CL is synthesized from PA via the cytidine diphosphate diacylglycerol pathway (40) and undergoes continuous membrane remodeling in the normal liver, whereas CL synthesis from PA is suppressed in metabolic dysfunction associated steatohepatitis livers subjected to IRI, leading to impaired mitochondrial function (41). PG is the direct precursor for CL synthesis mediated by cardiolipin synthase in the mitochondrial inner membrane. Therefore, a reduction in PG implies decreased CL biosynthesis (42). Consistent with this concept, in the CS group, PA (18:0/18:1), PA (18:0/20:4), and CL (74:7) were diminished, whereas these species were preserved in the MP-H2 group. Moreover, LPG (18:2) was reduced in the MP-H2 group (vs. MP and CS), providing the first demonstration of mitochondrial lipid remodeling during liver perfusion.

MP-H2 treatment resulted in the least lipid variability among groups, though saturated LPI species such as LPI 16:0, LPI 17:0, and LPI 18:0 remained elevated relative to controls. Since saturated fatty acids in Lyso-PLs occupy the sn-1 position and polyunsaturated fatty acids are predominantly bound at sn-2 (43), the increase in saturated LPI species suggests PLA2-mediated hydrolysis of PI (44). This implies that incomplete suppression of PLA2 activity in MP-H2 may contribute to persistent lipid remodeling and partial hepatic recovery failure. Compared with MP, MP-H2 showed higher PA levels but lower Lyso-PLs (particularly LPI) and FFAs. As PA serves as a key intermediate in PL and triacylglycerol synthesis (45) and increases during hepatic repair (46), its elevation indicates enhanced lipid metabolism induced by hydrogen supplementation. Lipid subclasses such as FFA, LPC, LPG, CL, MLCL, and DAG were significantly altered only in the CS group, demonstrating that MP effectively suppressed ischemic lipid degradation. Elevated DAG after reperfusion has been linked to increased ROS production via protein kinase C activation (47, 48). Therefore, its reduction in MP and MP-H2 likely reflects improved oxidative balance and lowered IRI severity. Hydrogen gas specifically influenced PA and LPI metabolism, distinguishing MP-H2 from MP. Meanwhile, Cer and LPE levels increased across all ischemic groups, consistent with previous findings linking their accumulation to apoptosis and impaired liver recovery (49). Cer serves as a branching point in the biosynthetic pathways leading not only to SM but also to various other sphingolipids, including ceramide 1-phosphate, glucosylceramide, and galactosylceramide (36). Further investigation is required to determine how the combined application of MP and hydrogen gas influences specific metabolic pathways of sphingolipids. Improvements in total MLCL/CL, LPC/PC, and LPG/PG ratios in MP and MP-H2 further indicate better maintenance of membrane lipids, while normalized LPI/PI, LPE/PE, and LPS/PS ratios in MP-H2 suggest mitigation of PL hydrolysis. Because elevated LPI/PI and LPE/PE ratios correlate with ischemia time and post-reperfusion injury (24, 25, 50), their stabilization highlights hydrogen’s role in preserving membrane integrity. The LPI/PI ratio reflected ischemic stress, consistent with previous reports (25). Moreover, maintaining the LPS/PS ratio may protect against IRI through MFGE8-mediated vesicular signaling (51).

Compared with the CS, both MP and MP-H2 treatments significantly reduced total PLA2 activity, indicating that HMP contributed to the suppression of PL hydrolysis. At the subclass level, however, the combination with hydrogen gas decreased total LPI, LPI/PI, and LPE/PE more markedly than HMP alone, suggesting that hydrogen supplementation facilitated membrane lipid remodeling. In cerebral IRI models, hydrogen has been reported to scavenge highly reactive radicals such as hydroxyl (OH) and peroxynitrite (ONOO^−^) (52). Moreover, hydrogen-rich preservation solutions have been shown to protect liver grafts by upregulating antioxidant enzymes, including heme oxygenase-1 (53). Several studies also suggest that hydrogen helps maintain intracellular calcium homeostasis (54, 55). Both oxidative stress and calcium overload promote the opening of mitochondrial permeability transition pores, leading to apoptosis (56); thus, hydrogen gas may protect mitochondrial membranes through its antioxidative activity and stabilization of calcium levels. Hydrogen gas–mediated protection of mitochondrial membranes may link to the remodeling of Lyso-PL/PL ratio. Although total PLA2 activity in the MP–H2 group was comparable to that in the control, total LPE and several LPI molecular species did not return to control levels. This discrepancy might be attributed to the substrate selectivity of individual PLA2 isoforms. The PLA2 superfamily comprises at least six major isozyme families with distinct substrate specificities (21). Identification of PLA2 isoforms that preferentially mediate the release of LPI and LPE, and administration of their selective inhibitors during liver transplantation could potentially enhance hepatic functional recovery. A principal limitation of this study is the lack of identification of key lipid-metabolizing enzymes, such as those involved in the Lands’ cycle and diacylglycerol kinase, which precludes a direct mechanistic interpretation of the lipidomic alterations. Nevertheless, the lipidomic profile strongly suggests a restoration of PL remodeling capacity in the MP-H2 group. In particular, the marked reduction in LPI may reflect suppression of PI-to-LPI conversion and/or enhanced reacylation toward intact PLs. In parallel, the decrease in DAG and recovery of PA imply restoration of DAG-to-PA flux, suggesting a transition from degradation-dominant lipid metabolism toward membrane repair–oriented remodeling.

Cold IRI has been consistently associated with enhanced PL deacylation and membrane degradation, as reflected by the accumulation of Lyso-PLs and FFAs (14, 57). Mechanistically, cPLA2 has been shown to hydrolyze mitochondrial membrane PLs, thereby promoting oxidative stress and apoptosis (24). In parallel, mitochondrial CL loss and oxidation are recognized hallmarks of IRI, contributing to the destabilization of mitochondrial cristae and respiratory dysfunction (58). Furthermore, recent imaging mass spectrometry studies have demonstrated that hepatic IRI induces depletion of PI accompanied by accumulation of LPL, indicating disruption of phosphoinositide remodeling (25). These findings collectively support a shift toward PL hydrolysis-dominant remodeling during cold IRI, reflecting an imbalance in Lands’ cycle between deacylation and reacylation processes. Consistent with this concept, our lipidomic analysis demonstrated marked accumulation of Lyso-PLs (LPI and LPC), FFA, DAG, and MLCL following CS and reperfusion, accompanied by a profound depletion of PA, indicating enhanced PL breakdown and impaired membrane remodeling. Importantly, these lipid alterations were observed in the same liver specimens in which we previously demonstrated increased hepatocellular injury, elevated apoptosis, and severe disruption of mitochondrial cristae structure after CS and reperfusion, whereas HMP partially preserved mitochondrial ultrastructure and hydrogen supplementation further reduced apoptosis and restored cristae density (13). These integrated findings strongly suggest that PL deacylation-driven membrane damage is closely linked to mitochondrial structural collapse and hepatocellular injury during cold IRI. The accumulation of MLCL observed in the present study is consistent with CL degradation and mitochondrial inner membrane destabilization, while the marked increase in LPI reflects disruption of phosphoinositide remodeling. Importantly, these lipid abnormalities were partially reversed by HMP and further normalized by HMP combined with hydrogen, with a particularly pronounced reduction in LPI. Given that HMP has been shown to restore mitochondrial redox balance and suppress reperfusion-associated oxidative stress (59), the observed normalization of Lyso-PLs and MLCL likely reflects attenuation of oxidative PL degradation at the mitochondrial membrane level. The additional effect of hydrogen is consistent with its ability to reduce oxidative stress and apoptosis and to preserve mitochondrial ultrastructure, as demonstrated in our previous study (13).

In summary, hydrogen-enriched hypothermic perfusion improved PI, PE, and PS metabolism, stabilized lipid remodeling, and reduced IRI-related damage. Importantly, this study is the first to demonstrate, to our knowledge, in HMP with hydrogen-supplementation, that LPI/PI, LPC/PC, and MLCL/CL sensitively reflect functional impairment in DCD rat livers. It also provides a comprehensive lipidomic profile of DCD rat livers under combined HMP and hydrogen treatment, offering new insight into lipid-mediated pathways in IRI. However, as the lipid content measurements were semiquantitative, further validation using clinically relevant models is warranted to clarify the functional roles of these lipid changes.

## Data Availability

The raw data are available from the corresponding author upon reasonable request.

## Supplemental Data

This article contains supplemental data.

## Conflict of Interest

The authors declare that they have no conflicts of interest with the contents of this article.

## Supplemental Data

Supplemental Table S1 A list of lipid metabolites and their relative concentrations.
